# Successful treatment with bortezomib for refractory fever associated with myelodysplastic syndrome with underlying lymphoplasmacytic lymphoma

**DOI:** 10.1002/ccr3.5372

**Published:** 2022-02-09

**Authors:** Yotaro Motomura, Yoshihiro Umezawa, Tomoyuki Arimatsu, Keigo Okada, Osamu Miura, Takashi Kumagai

**Affiliations:** ^1^ 26842 Department of Hematology Ome Municipal General Hospital Tokyo Japan; ^2^ 13100 Department of Hematology Tokyo Medical and Dental University Tokyo Japan

**Keywords:** Lymphoplasmacytic lymphoma/Waldenström's macroglobulinemia, myelodysplastic syndrome, myeloid differentiation primary response 88

## Abstract

We report a case of fever of unknown origin in a patient with MDS associated with IgM‐MGUS. The patient was positive for MYD88 mutation, and chemotherapy for LPL/WM improved the fever. Analysis of MYD88 and the effect of chemotherapy on LPL/WM finally revealed the latent LPL/WM in this case.

## Graphical abstract

We report a case of fever of unknown origin in a patient with MDS associated with IgM‐MGUS whose IgM level and rate of tumor cells were low. A positive MYD88 mutation and the effect of chemotherapy on LPL/WM finally revealed the latent LPL/WM.

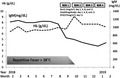

## 1 INTRODUCTION

Patients with myelodysplastic syndrome (MDS) experience fever caused by infection associated with febrile neutropenia and tumor fever due to progression to acute leukemia. Approximately 10%–30% of MDS cases have immune system disorders leading to fever.^1^

Lymphoplasmacytic lymphoma (LPL) is a lymphoid malignancy with immunoglobulin M (IgM) monoclonal gammopathy comprising minute monoclonal B cells, including mature B cells, differentiating into plasma cells. LPL with bone marrow invasion is defined as Waldenström's macroglobulinemia (WM).^2^ In the 2008 World Health Organization classification, characteristic cytogenetic and chromosomal abnormalities for LPL/WM were unclear; in a 2003 study, however, ~90% of LPL/WM cases had L265P mutation in MYD88 (myeloid differentiation primary response gene 88). The rate of malignant cells in the bone marrow of patients with WM has not been clearly defined.^2^ The pathological diagnosis for WM is occasionally difficult if the tumor burden is low in the bone marrow without extramedullary disease.

We have encountered an MDS case with recurring fever of unknown origin. No evidence of infection, tumor fever, or immunological diseases was found via various examinations. Bone marrow aspiration was consistent with low‐risk MDS as observed at diagnosis. Monoclonal IgM was positive but at low levels, consistent with monoclonal gammopathy of undetermined significance (MGUS).

The positive MYD88L265P mutation and good response to LPL/WM‐targeted therapy were useful for diagnosing underlying LPL/WM, a rare complication of MDS.

## 2 CASE REPORT

**FIGURE 1:**
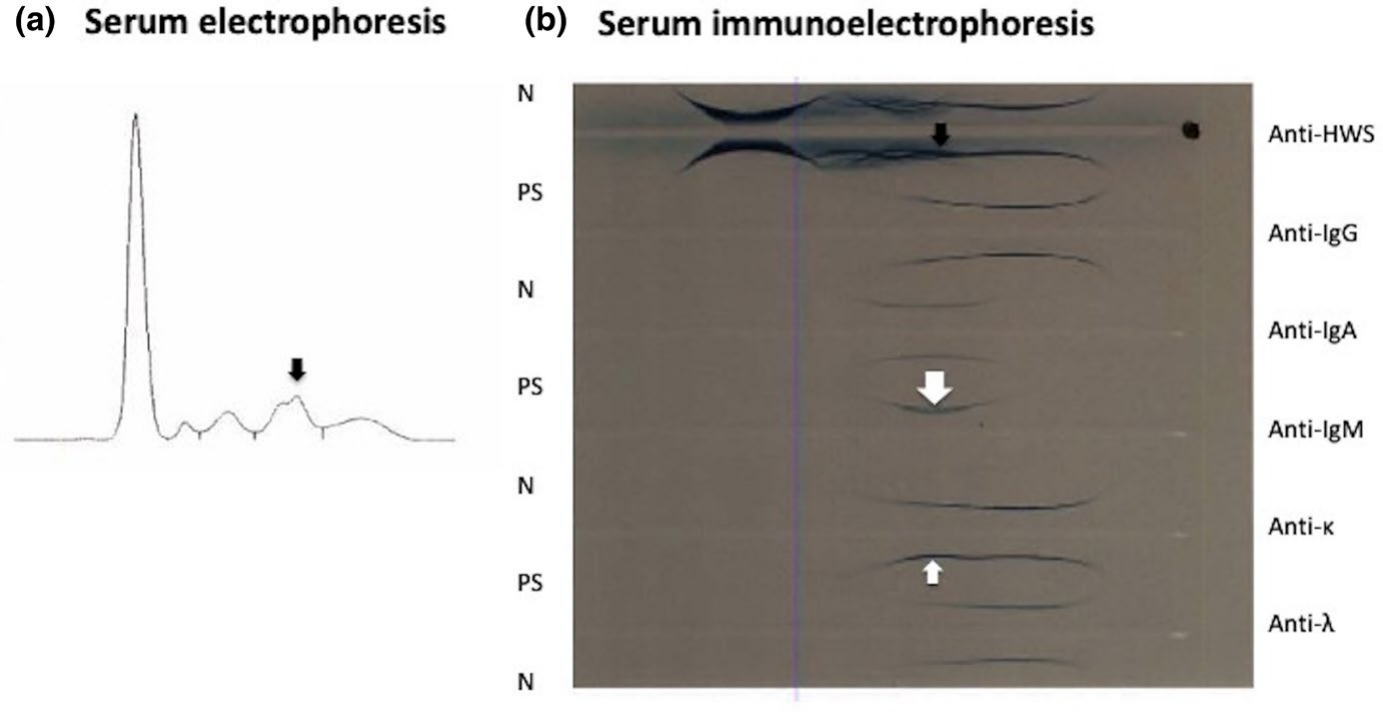
Serum protein electrophoresis and serum immunoelectrophoresis at diagnosis of MDS and IgM‐MGUS. a, Serum electrophoresis at diagnosis of MDS and IgM‐MGUS. A small M‐peak was seen (black arrow). b, Serum immunoelectrophoresis at diagnosis of MDS and IgM‐MGUS. An M‐bow was observed in response to anti‐IgM antibody (white arrow), and anti‐κ antibody (white smaller arrow), indicating the presence of monoclonal IgM‐κM protein. An M‐bow was also observed in response to anti‐HWS serum (black arrow). N: normal serum; PS: patient's serum; HWS: human whole serum

**FIGURE 2:**
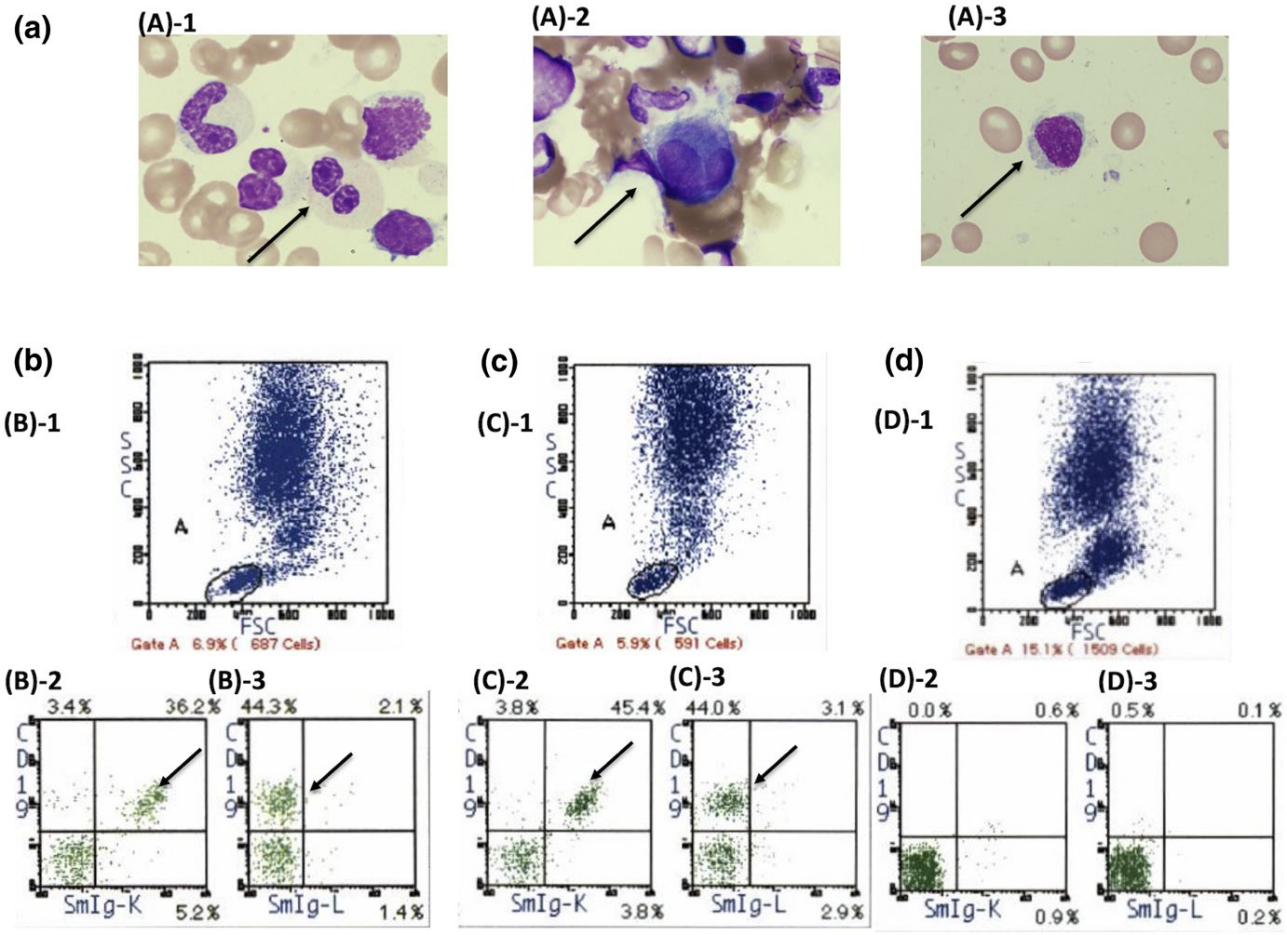
Bone marrow examination at repetitive fever onset (Aug 2, 2018). a, Bone marrow smear (Wright–Giemsa staining, ×1,000). MDS‐specific dysplasia including pseudo‐Pelger–Huët anomaly (A‐1, arrow) and micro‐megakaryocytes (A‐2, arrow) and abnormal lymphocytes (A‐3, arrow) were present. b, c, d, Flow cytometry at diagnosis of MDS (b, Dec. 15, 2016), before BDR therapy (c, Aug. 2, 2018) and after BDR therapy (d, Jan. 31, 2019). Flow cytometry revealed that 2.5% (B‐2 and B‐3, arrow) and 2.7% (C‐2 and C‐3, arrow) of nucleated cells were monoclonal B cells. FSC: forward scatter, SSC: side scatter

A 55‐year‐old man was diagnosed with classical Hodgkin's lymphoma and achieved complete remission by chemotherapy. At the age of 60, he was diagnosed with left renal cell carcinoma and achieved remission by left kidney resection. In November of 2016, at the age of 73, the patient was hospitalized for pneumonia and determined to have anemia. During the same hospital stay, he was also diagnosed with treatment‐related MDS (also known as MDS with multilineage dysplasia; International Prognostic Scoring System: low risk) by bone marrow aspiration, and with IgM‐MGUS by serum protein electrophoresis and immunoelectrophoresis (Figure 1). His marrow cells contained clonal B cells (Figure 2B and Table 1).

**TABLE 1:** Laboratory data at diagnosis of MDS, on admission and after BDR therapy

[Complete blood count]					[Serology Immunology]					[Bone marrow]				
	Dec.15, 2016	Aug.2, 2018	Jan.31, 2019			Dec.15, 2016	Aug.2, 2018	Jan.31, 2019			Dec.15, 2016	Aug.2, 2018	Jan.31, 2019	
WBC	3230	2720	2400	/μl	CRP	0.55	6.04	1.22	mg/dl	Ncc	9.2	18.6	14.5	×10^4^/μl
Neut	22.4	16.6	10.6	%	IgG	1025	786	339	mg/dl	MegK	38	6	25	/μl
Lym	44.7	12.7	60.7	%	IgA	204	147	50	mg/dl	M‐bl	4	3.6	2.2	%
Mon	32	69.3	27.3	%	IgM	951	1113	532	mg/dl	Promyel	1.1	2.1	1.1	%
Eos	0.7	0	0.7	%	sIL−2R	899	1484	1460	IU/ml	Myel	14.4	22	18.4	%
Bas	2	0	0.7	%	Β−2 MG	‐	16.7	11.3	mg/L	Metamyel	23.3	21	22.9	%
Atyp. Lym	0.4	1.3	0	%	WT1 mRNA	<50	<50	120	Copy/μg RNA	Stab	20.7	18.8	19.2	%
Hb	8.4	6.5	8.9	g/dl	[Chemistry]					Seg	15.5	9	10.2	%
MCV	118	108	122	Mm^3^	T‐Bil	0.3	0.3	0.4	mg/dl	Eos	0.1	0.3	0.4	%
Ret	2.7	1	1.3	‰	AST	19	57	17	IU/L	Bas	0.2	0	0.2	%
Plt	14.9	22.2	12.1	×10^4^/μl	ALT	23	59	20	IU/L	Lym	9.9	12.8	12.6	%
					γ‐GTP	67	109	135	IU/L	Mon	1.9	2.1	1.8	%
					LDH	148	220	223	IU/L	Plasma	1.2	1.2	0.2	%
					Alb	3.7	3	3.7	g/ml	Erythroid	8.2	1.6	8.0	%
					UA	6	6.7	4.7	mg/dl	M/E ratio	9.18	45.75	9.33	
					BUN	19.7	34.5	21.8	mg/dl	Atyp. lym	0.2	3.2	1	%
					Cre	1.17	4.24	2.89	mg/dl	Abnormal. Lym	0.1	2.4	1.3	%

In August of 2017, the patient experienced a transient fever of over 38°C. Because his white blood cell and neutrophil counts were 1,430 and 286/µl, respectively, the fever was considered febrile neutropenia. Renal dysfunction progressed because the patient had only one kidney (Table 1). No infections were detected by physical examination, various cultures, or fluorodeoxyglucose‐positron emission tomography (FDG‐PET)/computed tomography (CT) or other imaging. Serum examinations for bacterial, fungal, and viral infections were nondiagnostic. Autoimmune antibodies, including antinuclear antibody, were negative. Beginning in February 2018, his fever began to repeat periodically. Antibiotics or low‐dose steroids were ineffective.

Bone marrow aspiration/biopsy revealed hypercellular bone marrow with dysplasia, including pseudo‐Pelger–Huët anomaly and micro‐megakaryocytes, compatible with MDS (Figure 2A). Bone marrow aspiration also revealed that 2.4% of lymphocytes were abnormal (Table 1), and flow cytometry showed that 2.7% of nucleated cells were clonal B cells (k>>L) (Figure 2B).

**FIGURE 3:**
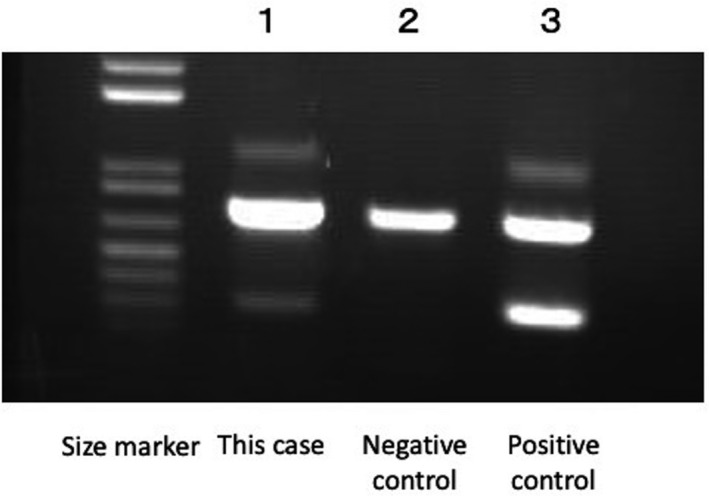
Analyses of MYD88L265P mutation using allele‐specific polymerase chain reaction (PCR). Genomic DNA from bone marrow mononuclear cells from the patient (lane 1) and those from patients negative and positive for MYD88L265P mutations (lanes 2–3) were analyzed at Tokyo Medical and Dental University using the allele‐specific polymerase chain reaction (PCR) method. The positions of the PCR products representing wild‐type MYD88 and MYD88L265P are illustrated

Allele‐specific polymerase chain reaction (PCR) for MYD88L265P using marrow cells was positive (Figure 3),^2^, ^3^ and no lymphadenopathy was observed on CT.

Based on the history of IgM‐MGUS, monoclonal B cells in the bone marrow, and positive MYD88L265P mutation, we considered that the tumor fever was due to LPL/WM developed from MGUS as a differential diagnosis.

Although MYD88 mutation has a high sensitivity for LPL/WM, its specificity is low. A search of the literature (Table 2)^4^, ^5^, ^6^, ^7^, ^8^, ^9^ revealed high MYD88‐positive rates in primary central nervous system lymphoma and testicular lymphoma. Therefore, other low‐grade B‐cell lymphomas could have the mutation. However, this case was clinically unlikely to be a primary central nervous system lymphoma, primary testicular lymphoma, or diffuse large B‐cell lymphoma based on PET/CT and repeat CT imaging studies.

**TABLE 2:** Lymphoma or IgM‐MGUS with high frequency of MYD88L265P mutation

Disease	Frequency	Reference
LPL/WM	91% (52/57)	Treon SP et al.(2012)^4^
LPL/WM	93% (97/104)	Xu L et al. (2013)^5^
LPL/WM	100% (58/58)	Varettoni M et al. (2013)^6^
IgM‐MGUS	10% (2/21)	Treon SP et al. (2012)^4^
IgM‐MGUS	54% (13/24)	Xu L et al. (2013)^5^
IgM‐MGUS	47% (36/77)	Varettoni M et al. (2013)^6^
PCNSL	72% (26/36)	Nayyar N et al. (2019)^7^
Primary testicular lymphoma	68% (25/37)	Kraan W et al. (2013)^8^
DLBCL (ABC type)	29% (13/45)	Ngo VN et al. (2011)^9^

**FIGURE 4:**
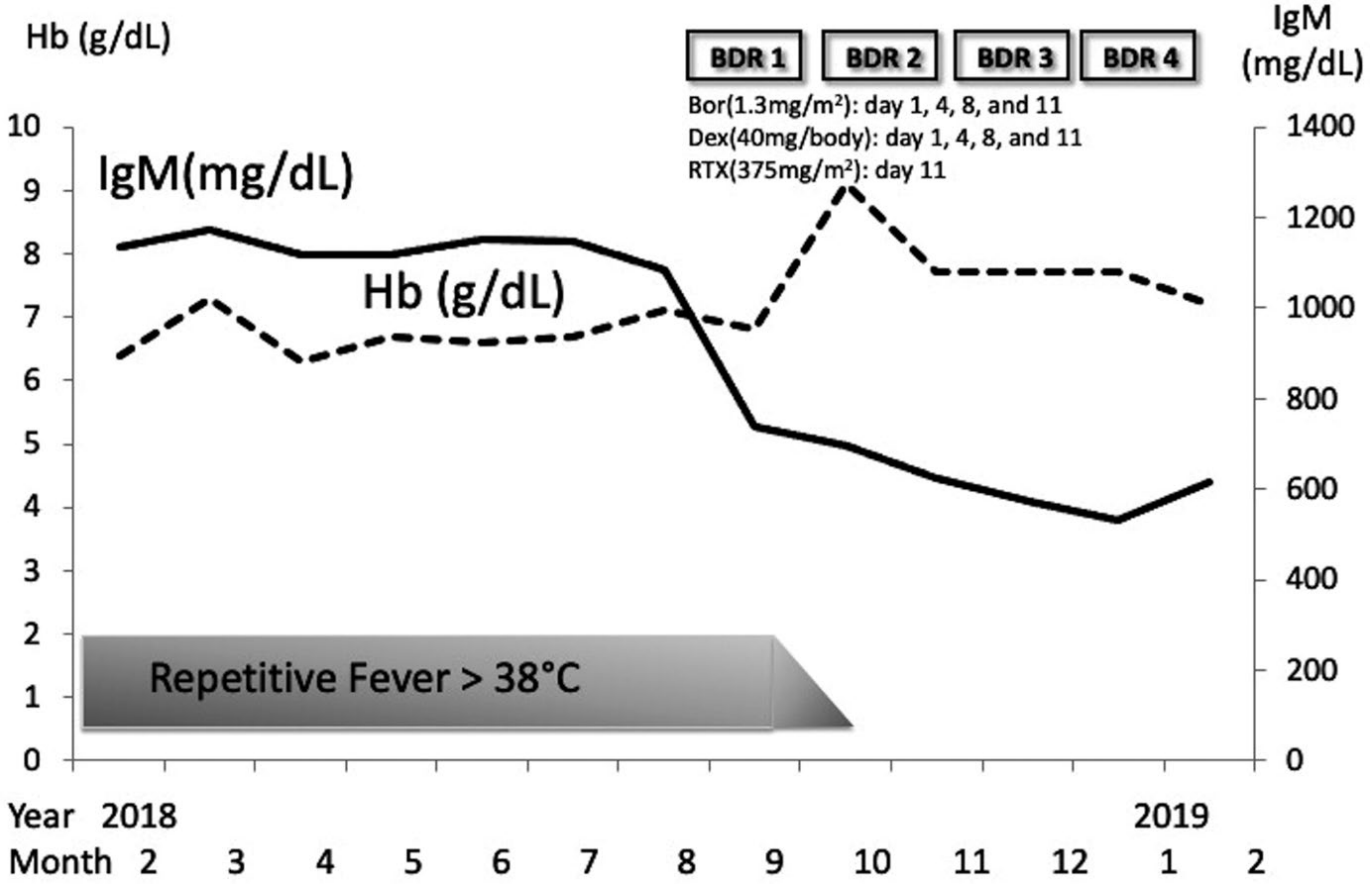
Clinical course of the patient. Bor, bortezomib; Dex, dexamethasone; RTX, rituximab

The MYD88‐positive rate is also high in IgM‐MGUS. We thus could not rule out IgM‐MGUS from LPL/WM. Therefore, in August of 2018, we administered bortezomib, dexamethasone, and rituximab (BDR) (bortezomib [1.3 mg]/m^2^ on days 1, 4, 8, and 11; dexamethasone [40 mg] /body on days 1, 4, 8, and 11; and rituximab 375 mg/m^2^ on day 11^10^) targeted for possible symptomatic LPL/WM. After four cycles of BDR, IgM M‐proteins decreased from 1113 mg/dl to 532 mg/dl (partial remission). The recurring fever over 38°C dramatically improved to a normal level. Flow cytometry showed that the clonal B cells that had been previously detected had almost completely disappeared (Figure 2B‐D). Also, the bone marrow aspiration showed that the number of abnormal lymphocytes was reduced to 1.3% (Table 1). BDR therapy was considered to have been successful (Figure 4).

## 3 DISCUSSION

In patients with MDS, infections, malignancies, or autoimmune diseases cause fever. In the patient in this case report, bone marrow examination with flow cytometry and chromosomal analysis did not reveal the diagnosis of LPL/WM.

In our patient, it was important to determine the cause of the increase in serum monoclonal IgM, because it could have been derived from various B‐cell malignancies other than LPL/WM.

For these reasons, MYD88 mutation analysis is useful for diagnosing LPL/WM. In our patient, the good response of the fever of unknown origin to BDR therapy confirmed the diagnosis.

MYD88L265P mutation may be an early oncogenic event in LPL/WM,^4^ and MYD88L265P‐positive IgM‐MGUS cases are more likely to progress to LPL/WM than MYD88L265P‐negative ones.^5^ Therefore, we should exercise caution regarding the malignant transformation of MYD88L265P‐positive IgM‐MGUS.

In a study involving 40 patients with MDS, stem cells were shown to overexpress the MYD88 protein. However, MYD88L265P was not found in all cases was not found in any cases.^11^ Therefore, MYD88L265P might be derived from LPL/WM but not MDS.

The International Workshop on Waldenström's Macroglobulinemia provides the criteria for starting treatment for LPL/WM.^12^ In this case, the recurring fever and anemia met the criteria for LPL/WM treatment despite low IgM levels. Although the anemia might have been derived from MDS, it improved after four cycles of BDR therapy, suggesting that LPL/WM caused both fever and anemia

Many rituximab‐containing regimens are effective for WM/LPL based on phase II trials. In this case, BDR was selectively administered because of the renal impairment (creatinine clearance rate: 18 ml/min/1.73 m^2^).

The prevalence of MGUS in patients with MDS ranges between 2% and 10%. Moreover, in a study on 1,198 patients with MDS, 6 patients had complications of LPL.^13^ This finding would suggest that MDS with associated IgM‐MGUS or LPL/WM is not especially rare.

Clinicians should watch for symptomatic LPL/WM in their patients with MDS, as in the present patient, whose IgM level and rate of tumor cells were low. In this patient, MYD88 mutation analysis and BDR therapy played a significant role in diagnosing LPL/WM and informing the treatment strategy.

## ACKNOWLEDGMENT

Not applicable.

## CONFLICT OF INTEREST

The authors declare no conflict of interest.

## AUTHOR CONTRIBUTIONS

Yotaro Motomura drafted the article and provided and cared for study patients. Yoshihiro Umezawa and Osamu Miura served as scientific advisors. Tomoyuki Arimatsu and Keigo Okada provided and cared for study patients. Takashi Kumagai critically revised the article for important intellectual content.

## CONSENT

Written informed consent was obtained from the patient to publish this report in accordance with the journal's patient consent policy.

## Data Availability

The data that support the findings of this study are available from the corresponding author upon reasonable request.
